# Helping the forcely displaced persons - situation in Hungary

**DOI:** 10.1192/j.eurpsy.2023.179

**Published:** 2023-07-19

**Authors:** G. T. Szekeres

**Affiliations:** Hungarian Psychiatric Association, Budapest, Hungary

## Abstract

**Abstract:**

In recent years, the state-funded side of the Hungarian psychiatric system has been struggling with a serious lack of resources. During the pandemic the level of organization, which was also not perfect, continued to deteriorate. Immediately after the breakout of Ukrainian war the civilians started to make significant efforts to support refugees. From the beginning our Association joined activities of Solidarity Network organized by EPA. We made efforts among other things to find Ukrainian-speaking psychiatrists to tackle the language barriers, connect the needs with the offers psychologists. When a refugee is admitted to a psychiatric unit as a patient, they receive all mental health care as any Hungarian citizen would. Overall however, mental care is insufficiently organized and the patient pathways for refugees - as those of the population - are not well-defined. The coordination between government services and civil organizations helping refugees is unsatisfactory. There is no steady support system that could provide regular, professional mental health care for those in need, thus a mental triage process is also missing to uncover the perhaps less severe mental problems, that nevertheless might require professional attention. Consequently there also seems to be a lack of assistance in trauma processing. There are some official civil organizations (e.g. Maltese Charity Service, Hungarian Red Cross) that are doing all they can for the refugees, but most of their staff is not professionally equipped to provide mental health care or to reliably identify when it is needed. When refugees show symptoms of a severe nature (psychosis, mania, severe depression, suicidal thoughts) they reach out to the professional mental health care system, but refugees struggling with less obvious mental health problems generally stay out of sight of professional psychiatric or psychological care.

**Keywords:**

civilian efforts, unmet needs, lack of human resource

**Disclosure of Interest:**

None Declared

